# Comparative study of conventional and synchrotron X-ray electron densities on molecular crystals

**DOI:** 10.1107/S2052520623006625

**Published:** 2023-09-04

**Authors:** Emilie S. Vosegaard, Jakob V. Ahlburg, Lennard Krause, Bo B. Iversen

**Affiliations:** a Center for Integrated Materials Research, Department of Chemistry and Interdisciplinary Nanoscience Center (iNANO), Aarhus University, Aarhus C, DK-8000, Denmark; Politecnico di Milano, Italy

**Keywords:** electron density, data quality, molecular crystal, single crystal X-ray diffraction

## Abstract

Five different electron density datasets obtained from conventional and synchrotron single crystal X-ray diffraction experiments are compared. The general aim of the study is to investigate the quality of data for electron density analysis from current state-of-the-art conventional sources, and to see how the data perform in comparison with high-quality synchrotron data. A molecular crystal of melamine was selected as the test compound due to its ability to form excellent single crystals, the light atom content, and an advantageous suitability factor of 3.6 for electron density modeling. These features make melamine an optimal system for conventional X-ray diffractometers since the inherent advantages of synchrotron sources such as short wavelength and high intensity are less critical in this case. Data were obtained at 100 K from new in-house diffractometers Rigaku Synergy-S (Mo and Ag source, HyPix100 detector) and Stoe Stadivari (Mo source, EIGER2 1M CdTe detector), and an older Oxford Diffraction Supernova (Mo source, Atlas CCD detector). The synchrotron data were obtained at 25 K from BL02B1 beamline at SPring-8 in Japan (λ = 0.2480 Å, Pilatus3 X 1M CdTe detector). The five datasets were compared on general quality parameters such as resolution, 〈*I*/σ〉, redundancy and *R* factors, as well as the more model specific fractal dimension plot and residual density maps. Comparison of the extracted electron densities reveals that all datasets can provide reliable multipole models, which overall convey similar chemical information. However, the new laboratory X-ray diffractometers with advanced pixel detector technology clearly measure data with significantly less noise and much higher reliability giving densities of higher quality, compared to the older instrument. The synchrotron data have higher resolution and lower measurement temperature, and they allow for finer details to be modeled (*e.g.* hydrogen κ parameters).

## Abstract 2

Four electron density quality single crystal X-ray diffraction datasets on molecular crystals of melamine were obtained from both state-of-the-art laboratory instruments (Rigaku Synergy with Ag *K*α and Mo *K*α, Stoe Stadivari with Mo *K*α) and an older X-ray diffractometer (Oxford Diffraction Supernova with Mo *K*α) and the data quality and electron density models were compared with results from a high-quality synchrotron dataset (SPring-8).

## 1. Introduction

Melamine, with the systematic name 1,3,5-triazine-2,4,6-tri­amino and chemical formula C_3_H_6_N_6_, crystallizes in the monoclinic space group *P*2_1_/*n*. Owing to the high electron density (ED) suitability factor of 3.6, light elements, and low symmetry, melamine is an ideal crystal for X-ray ED analysis (Coppens, 1997 ▸; Stevens & Coppens, 1976 ▸). Here we compare different datasets and the resulting ED models to probe the experimental requirements for obtaining data of high enough quality for reliable ED determination of a small organic molecule like melamine. In order to investigate the effect of different X-ray sources we compare single crystal X-ray diffraction data obtained from the BL02B1 beamline at the SPring-8 synchrotron facility in Japan to different conventional sources available in-house at Aarhus University. The test system was chosen to be a simple case favoring the conventional sources, and results presented here therefore cannot generally be used to infer how well conventional X-ray sources perform for less suitable samples (poor scattering, heavy atoms, anharmonicity, absorption and extinction effects).

Similar comparisons of instrumentation have been performed with the same goal to assess the challenges and opportunities when measuring ED quality data (Kamiński *et al.*, 2014 ▸; Martin & Pinkerton, 1998 ▸; Wolf *et al.*, 2015 ▸; Herbst-Irmer & Stalke, 2017 ▸). Previous data comparison studies from our group (Schmøkel *et al.*, 2013*a* ▸,*b* ▸; Tolborg & Iversen, 2019 ▸) concerned mainly inorganic materials, imposing a variety of restriction on the instrumentation needed for high quality data. Schmøkel *et al.* (2013*b* ▸) and Jørgensen *et al.* (2014 ▸) emphasize how the short wavelength, low temperature, and the high intensity obtainable at synchrotrons are necessary for successful deconvolution of thermal and electronic effects for the low valence-to-core electron ratio for samples with heavier elements, and to limit extinction and absorption effects. For organic molecular crystals problems with absorption and low valence-to-core electron ratio are usually not a concern. However, the intrinsically lower scattering power of lighter elements imposes requirements on the intensity of the X-ray beam. Usually due to the *I* ∝ λ^3^ dependence of the diffracted beam, a longer wavelength, like Cu radiation with a wavelength of 1.54 Å, is desired at conventional sources for organic samples. A longer wavelength unfortunately also limits the data resolution, and for the optimal access to a large portion of reciprocal space a Mo source with a wavelength of 0.71 Å is typically chosen for ED measurements on organic compounds. This makes it possible to reach a high resolution of ∼1.25 Å^−1^ (0.4 Å) within a reasonable time frame. In the present case an Ag source is also available, but even though the lower wavelength of 0.56 Å collapses reciprocal space up to a resolution of 1.25 Å^−1^ onto a single detector position, the need for a much higher exposure time due to the lower intensity makes the total collection time significantly longer. The higher intensity at the synchrotron source makes it possible to collect data at an even shorter wavelength increasing the maximum resolution of the dataset. The lowest possible temperature and highest possible resolution are desirable for accurate determination of vibrational parameters (Iversen *et al.*, 1996 ▸). The very high maximum resolution of 1.45 Å^−1^ obtained for the system at the synchrotron source is the foundation for an accurate determination of the thermal motion of non-hydrogen atoms, since at this high resolution only scattering from core electrons is present.

We recently published an elaborate description of chemical bonding in melamine based on analysis of the experimental ED obtained from the X-ray diffraction experiment performed at 25 K at the SPring-8 synchrotron (Vosegaard *et al.*, 2022 ▸). From this analysis we found that a range of weaker intermolecular interactions, mainly π–π interactions and hydrogen bonding govern the crystal structure. Concerning chemical conclusions, we refer to that study, and here we instead focus primarily on the experimental and crystallographic analysis of melamine in a broader perspective. We present four multipole models and the resulting experimental EDs based on 100 K datasets obtained from in-house diffractometers Rigaku Synergy-S, Stoe Stadivari and an older Oxford Diffraction Supernova. The Synergy diffractometer has a dual Mo and Ag source, while datasets from the two other conventional diffractometers were obtained with Mo sources. The Stadivari diffractometer is equipped with a Cu source in addition to the Mo source, but due to the restricted maximum resolution available, a dataset from the Cu source was not obtained for this study. The conventional datasets are benchmarked against the published 25 K high-quality synchrotron dataset obtained at the BL02B1 beamline at SPring-8.

## 2. Experimental

Five different experimental single crystal X-ray diffraction datasets are compared, one synchrotron dataset from SPring-8 collected at 25 K, one from the Stoe Stadivari and one from an Oxford Diffraction Supernova using Mo sources, and two from the Rigaku Synergy-S with Ag and Mo sources, respectively.

The synchrotron data were measured at the BL02B1 beamline at SPring-8 in Japan. The beamline is equipped with a quarter χ goniometer and a Pilatus3 X 1M CdTe detector. An X-ray energy of 50 keV, corresponding to a wavelength of 0.248 Å, was used. The Stadivari and Synergy-S diffractometers are both new instruments at the Department of Chemistry at Aarhus University. The Stadivari is equipped with a PRIMUX 50 microfocus Mo *K*α X-ray source system, a quarter χ goniometer and an Eiger2 R 1M CdTe detector, while the Synergy has a PhotonJet-S microfocus X-ray source, a κ goniometer and a HyPix-arc 100° detector with two non-parallel modules to cover a larger part of reciprocal space. Both the Eiger2 R 1M CdTe and the HyPix-arc 100° are Hybrid Photon Counting (HPC) detectors, but where the EIGER2 1M has a cadmium telluride sensor, the HyPix-arc 100° sensors are made of silicon. The Supernova is an older diffractometer (∼12 years) with κ geometry and an Atlas Charge Coupled Device (CCD) detector. All in-house diffractometers are equipped with Oxford Cryostream 800 liquid nitro­gen cryostats, allowing for data collection at 100 K. The synchrotron dataset was obtained at 25 K with a liquid He jet stream. The lowest possible temperature was used to enhance the signal-to-noise level, while reducing thermal diffuse scattering and providing better estimates of the vibrational parameters. In the following figures and tables, the five different datasets will be denoted: SPring-8, Stadivari, Supernova, Synergy_Ag (Syn_Ag) and Synergy_Mo (Syn_Mo).

Single crystals were prepared following procedures described by Vosegaard *et al.* (2022 ▸). Three different crystals from the same batch were used for the synchrotron and in-house measurements, respectively. Two larger crystals (Fig. S1 in the supporting information) with dimensions (µm) of 200 × 160 × 100 and 190 × 140 × 100 were selected and used for the conventional sources to reduce the overall collection time. The full width at half-maximum (FWHM) of the beam for all conventional sources is smaller than the crystals, causing different sample volumes to be probed by the beam at different orientations of the crystal, but due to the low absorption of the crystal and the large data redundancy, we expect no significant effect from this. A smaller crystal 140 × 100 × 100 µm was used at SPring-8, where the high intensity makes data collection fast. We used the optimal run strategy suggested by the instrument with user preferences of mean intensity divided by standard uncertainty 〈*I*/σ〉 = 15, redundancy = 7 and a resolution of *d* = 0.4 Å (corresponding to sinθ/λ = 1.25 Å^−1^) for all in-house diffractometers. For datasets with Mo sources, this meant having different exposure times for low and high detector angles to reduce collection time, while obtaining a satisfactory intensity for the high angle data. As can be seen in Table 1 ▸, the necessary collection times differ significantly from instrument to instrument based on mainly beam intensity, detector efficiency and motor velocity. The synchrotron data collection was remarkably faster than the in-house experiments. For the in-house datasets the detector distances were generally chosen to be the lowest possible distance to reduce data collection times, while still resolving individual peaks. For the Stadivari, the detector distance was set to 60 mm to avoid a shadow from the cooling nozzle on the detector frames and the maximum resolution was limited to 1.17 Å^−1^ due to restrictions on the motor movements to avoid the collision of equipment. For the Supernova, the maximum resolution of 1.17 Å^−1^ stated in Table 1 ▸ was selected after attempts to use data up to 1.25 Å^−1^ gave poor results (this will be discussed later). The experimental strategies, including run lists, can be found in supporting information.

The data were initially evaluated through a set of global quality parameters such as the internal reliability factor, *R*_int_, and the completeness, as seen in Table 1 ▸. *R*_int_ should be as low as possible while the completeness should ideally be 100%, as we see for all datasets in this study. *R*_int_ is well below 5%, except for the Supernova dataset, which has a higher *R*_int_ (also discussed later).

In the case of multipolar refinements, the mean intensity divided by the standard uncertainty, 〈*I*/σ〉, the redundancy and the number of measured reflections, *N*, also play an important role. These parameters can be seen in Table 1 ▸. All three values vary with the resolution, and it is generally expected that the data quality and signal-to-noise ratio are lower for reflections measured at high sinθ/λ (low *d*-spacing), and for a fair comparison of the five datasets, the 〈*I*/σ〉 and redundancies are reported only to the highest common sinθ/λ value of 1.17 Å^−1^. The redundancy is a measure of how many times each reflection is measured on average. The higher the redundancy, the better estimate of uncertainties. 〈*I*/σ〉 is a measure of the significance and reliability of each intensity. Finally, the number of measured reflections reflects the initial amount of data that has gone into the data treatment procedure. *N*_unique_ is the number of unique reflections measured more than three times, which is also the number of structure factors included in the actual multipole model refinement. For the multipole model where a large number of parameters are introduced to refine the aspherical electron densities on each atom, many reflections are necessary for a statistically significant result.

For all reported datasets the redundancy is close to or above 7 and the 〈*I*/σ〉 values are between 30 and 40, except for those collected with the Supernova (see below). The number of unique reflections for the in-house datasets are 6000–8000, and much higher (above 13000) for the synchrotron dataset as is to be expected due to the higher intensity, shorter wavelength and larger angular resolution.

### 2.1. Data integration procedure

Integration procedures depend on the instrument and the corresponding software. The initial peak hunting and integration were performed in the software provided with the instrument for the in-house measurements. For the synchrotron experiment the data treatment procedure followed the same steps as described by Vosegaard *et al.* (2022 ▸), where *SAINT-Plus* (Bruker, 2012 ▸) was used for integration. Integration options included least-squares profile fitting and recurrence background subtraction, with an active image queue half-width of 25 images. Absorption correction and scaling was performed in *SADABS* (Krause *et al.*, 2015 ▸).

The two Synergy-S datasets and the Supernova dataset were all reduced using the program *CrysAlisPro*. A smart background was used with a frame range of 15, and outlier rejection was performed based on the 2/*m* Laue group. For all datasets a multi-scan absorption correction with a subsequent μ_r_ spherical correction, to account for the angular part, was applied. Finally, an error model was applied to account for machine errors (error model 1; *CrysAlisPro*), and in the case of the Supernova dataset, also a model to separate gains for signal and background counts (error model 5; *CrysAlisPro*).

Data integration for the Stadivari dataset was performed with the *X-AREA* (Stoe & Cie, 2016 ▸) software. Instrument specific corrections were accounted for automatically. A, B and EMS parameters used for the integration were obtained from the optimization procedure to 9.0, −1.0 and 0.008, respectively. For the final scaling, third-degree polynomials were used for both frame scaling and *x*,*y* detector scaling, while beam(in) and beam(out) scaling was performed with *l*(max) of 4 and 1 for the even and odd harmonics, respectively. Outlier rejection was based on the monoclinic 2/*m* Laue class and a spherical absorption correction was applied according to the crystal size and wavelength.

Further merging and estimation of uncertainties was performed in *SORTAV* (Blessing, 1997 ▸) for all datasets.

### 2.2. Structure solution and multipolar refinements

The procedure for structure solution and refinements performed for all datasets in this study follows the same routine reported in detail by Vosegaard *et al.* (2022 ▸), so it will only be shortly summarized here. All structures were solved and refined in *OLEX2* (Dolomanov *et al.*, 2009 ▸) using *SHELXT *(Sheldrick, 2008 ▸, 2015*a* ▸) and *SHELXL* (Sheldrick, 2015*b* ▸), respectively. Hydrogen bond lengths and anisotropic atomic displacement parameters (ADPs) were obtained from Hirshfeld Atom Refinement (HAR) (Capelli *et al.*, 2014 ▸; Fugel *et al.*, 2018 ▸) of the experimental geometry using *Tonto* with a B3LYP/def2-SVP method/basis set and an 8 Å cluster in *NoSpherA2* (Kleemiss *et al.*, 2021 ▸). The ED was refined using the Hansen–Coppens multipolar formalism (Hansen & Coppens, 1978 ▸) in *XD2016* (Volkov *et al.*, 2016 ▸) with the Su, Coppens, Macchi radial function databank (Su & Coppens, 1998 ▸, Macchi & Coppens, 2001 ▸). All multipoles up to the hexadecapole level (*l* = 4) were refined for the *p*-block atoms, while only bond directed dipoles and quadrupoles were refined for hydrogen. Atoms with similar chemical environment were given the same κ and κ′ parameters. For the conventional sources, the multipole model was unable to converge when κ on hydrogen atoms were refined, so hydrogen κ parameters were set to 1.15 and 1.4 for spherical κ and κ′, respectively (Volkov *et al.*, 2001 ▸). For the synchrotron dataset, it was possible to refine the hydrogen κ parameters to values of 1.139 (7) and 1.388 (24) for κ and κ′, respectively, a feature attributed to the higher data quality (higher maximum resolution and lower temperature). The values are very similar to the tabulated values, supporting the choice of κ values for the conventional sources. For a better basis for the comparison, all datasets reported here were refined using fixed κ values.

The naming convention of the atoms in the melamine molecule used throughout this paper can be seen in Scheme 1. 
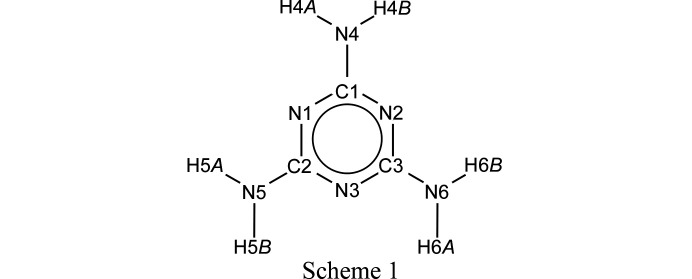


## 3. Comparison

The quality of the experimental data is based on several different parameters and it has a significant impact on the final results. In the following sections a comparison of the data quality and model results is performed. Assessment of data quality includes evaluation of the observed and measured intensities, as well as a comparison of the quality parameters and plots from the multipolar model. For analysis of the data, the resulting model and the described electron density, different key parameters are compared, including unit-cell parameters, multipole populations and topology.

### 3.1. Structure factors

**Figure 1:**
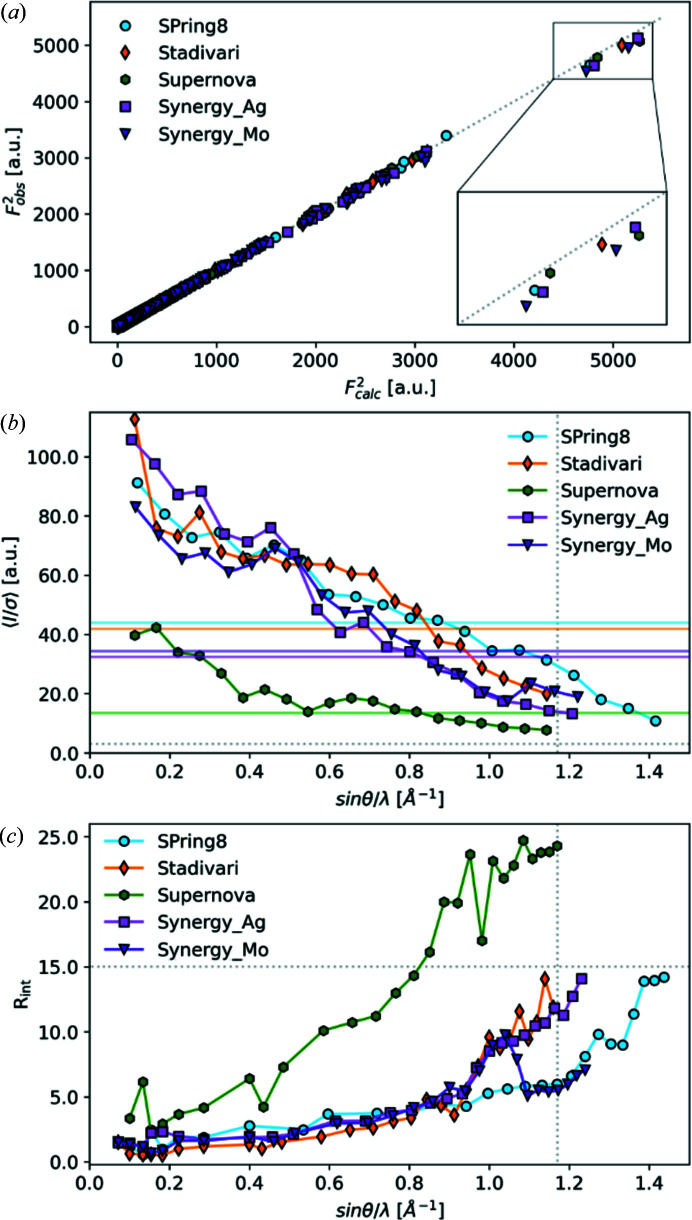
(*a*) Observed structure factors squared 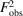 shown as a function of calculated structure factors squared 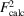 . The gray dotted line shows the ideal correlation of 1. (*b*) Mean intensity divided by the standard uncertainty of reflections 〈*I*/σ〉. Dotted gray horizontal and vertical lines show the *I* > 3σ intensity cutoff used in the multipole model and the 1.17 Å^−1^ resolution cutoff used in some of the comparisons in this paper. Colored lines show the average 〈*I*/σ〉 of each dataset for all reflections. (*c*) Merging *R* factor, *R*_int_, as a function of resolution. Dotted gray horizontal and vertical lines show the 15% quality line and the 1.17 Å^−1^ resolution cutoff used for comparisons.

Comparison of structure factors obtained from different experiments will show systematic tendencies and instrument-specific flaws.


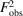
 as a function of 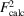 follows a linear trend almost equal to 1 for all datasets as seen in Fig. 1 ▸(*a*). Curiously, most datasets seem to underestimate the observed intensity for the strongest reflections [insert in Fig. 1 ▸(*a*)] compared to the model, a problem well known for the Pilatus detector at the BL02B1 beamline at SPring-8 (Krause *et al.*, 2020 ▸), but not commonly encountered for conventional sources. The SPring-8 dataset was measured with no attenuation, resulting in a maximum flux significantly outside of the linear detection range of the detector. This was expected to result in underestimation of the most intense reflections as observed, but seems to have no impact on the model.

Fig. 1 ▸(*b*) shows that for all datasets the mean intensity divided by uncertainty, 〈*I*/σ〉, is significantly higher than the *I* > 3σ intensity cutoff used for the final model. The horizontal lines show the average of each dataset up to a resolution of 1.17 Å^−1^ that can also be seen in Table 1 ▸. As expected, the synchrotron dataset has more significant reflections (a higher 〈*I*/σ〉) than the others, but for the low-angle data (< 0.5 Å^−1^) the three conventional datasets from the new diffractometers are similar to the SPring-8 in significance. The Supernova dataset has a remarkably lower 〈*I*/σ〉 for the full resolution range, but still lies significantly above the *I* > 3σ intensity cutoff. The excellent 〈*I*/σ〉 for the new instruments is attributed to the virtually noise free photon counting detectors with very low background, while the lower 〈*I*/σ〉 for the Supernova is believed to stem from intrinsic detector noise and degradation of the older CCD detector.

To assess the success of the integration procedure and the equivalence of symmetry related reflections, the merging *R* factor, *R*_int_, is shown in Fig. 1 ▸(*c*) as a function of sinθ/λ. A low *R*_int_ is most important for the low angle data in ED analysis, as this is where the valence electron scattering is present. For all datasets we observe an increase in *R*_int_ with scattering angle, but for the synchrotron and new in-house diffractometer datasets it stays below 15% for the full resolution range. A higher *R*_int_ was expected for the Supernova dataset, but above 0.8 Å^−1^ *R*_int_ actually increases to more than 20%. The total *R*_int_ has a reasonable value of 10.5%, and the maximum resolution of 1.17 Å^−1^ is maintained for the Supernova dataset.

### 3.2. Quality of the multipolar model

For multipole models obtained from different datasets we expect no deviations due to the difference in temperature, since in the ideal case this should only affect the atomic displacement parameters, and not the modeling of the valence electron density. All datasets provide reliable multipole models of excellent quality based on data measured to high resolution (>1.17 Å^−1^), with low *R* factors (<3.5%) and low min/max residuals (< ±0.3 e Å^−3^) as seen in Tables 1 ▸ and 2 ▸.

**Figure 2:**
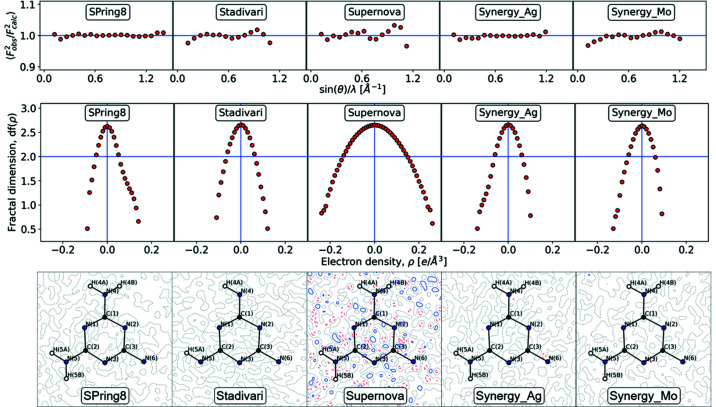
Top: Observed structure factor squared 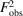 divided by calculated structure factor squared 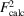 , plotted in bins from 0 to 1.5 Å^−1^ (Zavodnik *et al.*, 1999 ▸, Zhurov *et al.*, 2008 ▸). Middle: Fractal dimension plots (Meindl & Henn, 2008 ▸) of all datasets calculated from a 1 × 1 × 1 unit-cell cube of the electron density with 72 × 80 × 108 grid points along the *a*, *b* and *c* direction, respectively, for a uniform grid. Bottom: Residual density maps in the plane of the ring. Blue means that a higher electron density is observed than calculated, while red is lower electron density. The black dotted line is zero. Contours are shown at 0.1 e Å^−3^ intervals. Only atoms within a 0.2 Å distance of the plane defined by C1/C2/C3 are labeled in the plot. Both the fractal dimension plots and residual density maps are calculated at the largest common resolution cutoff at 1.17 Å^−1^ for a better comparison.

The upper figure panel in Fig. 2 ▸ shows binned 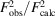 as a function of resolution. Binning the data removes random deviations present at especially high sinθ/λ. For a perfect model ( 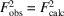 ) the red dots will fall on the blue line for the full resolution range. Deviations are often seen at low angles due to low statistics (few very intense, possibly underestimated reflections in each bin, as apparent from Fig. 1 ▸), but systematic trends like extinction should also become apparent. For all datasets we find an acceptable agreement between observation and calculation, with the Spring 8 data being the best and the Supernova data being somewhat worse at high resolution, although still within a reasonable margin of ±5%. It could be argued that the Supernova data should have been cut at even lower resolution to avoid noise in the higher order data, but even though the data has lower quality than the other datasets, it still provides a reliable ED model.

The fractal dimension plots in the middle panel of Fig. 2 ▸ should be parabolic in shape, and as narrow as possible, to signify random errors and small residuals in the ED. In all cases the fractal dimension plots are very narrow suggesting very low min/max residuals as can also be seen in Table 2 ▸ and the bottom panel of Fig. 2 ▸. A shoulder to the positive side can be seen for the synchrotron dataset suggesting some slight systematic errors in the model. The Supernova fractal dimension is very parabolic, suggesting only random errors, but it is much wider than the others. It may be that the larger random errors hide smaller systematic error in the model.

Residual density maps in the bottom panel of Fig. 2 ▸ are very flat (indicating low max/min residuals) with virtually no features for most cases. The largest residuals are seen for the Supernova as also noted earlier, but the residual density appears to have a random nature, supported by the parabolic shape of the fractal dimension plot, so it should have low impact on the final model.

The number of reflections included in the model may have a significant impact on the reliability of the results. *N*_ref_ seen in Table 2 ▸ is the number of reflections used for the refinements and is lower compared to *N*_unique_ seen in Table 1 ▸ due to a *I* > 3σ filtering of the observed reflections. Especially for the Supernova dataset a large part of the measured structure factors are discarded due to this criteria as can be seen from the higher *N*_rejected_. Quality parameters are also reported for the full dataset with no rejection criterion (no *I*/σ cutoff), showing only a slight decrease in model quality compared to the 3σ criterion used in the reported model. A comparison of quadrupole parameters and a difference density map is presented in supporting information. This serves as evidence of very robust models. Only the Supernova dataset shows a significant increase in *R* factors and residuals. To report only the most significant data, the *I* > 3σ cutoff was applied for the models reported here. The number of included reflections divided by the number of variables refined, *N*_v_, in the model, *N*_ref_/*N*_v_, should be as high as possible for reliable determination of the refined parameters in the least-squares procedure. For all datasets the *N*_ref_/*N*_v_ is larger than 10 as seen in Table 2 ▸, and the models can generally be considered to be of high quality. The number of reflections included in the comparisons to 1.17 Å^−1^ (*N*_ref_) shows that even though the datasets are compared at a similar maximum resolution, the structure factor lists are not the same, and the number of reflections for the synchrotron dataset is still significantly higher than the in-house datasets with a factor of 1.1 reflections more than the Synergy-S datasets (corresponding to approx. 800 reflections), 1.3 for the Stadivari dataset (1538 reflections) and 1.5 for the Supernova dataset (2172 reflections). The correlation coefficient, also seen in Table 2 ▸, quantifies the relationship between correlated parameters. For the SPring-8 dataset the most correlated pair is the κ-parameter on C atoms and the monopole on C2, having a correlation coefficient of 0.77. Generally all observed correlations are explained by the intrinsic nature of the multipole model, and with relatively low correlation coefficients below 0.80, no systematic flaws in the models are deduced from this parameter.

### 3.3. Unit-cell parameters

**Figure 3:**
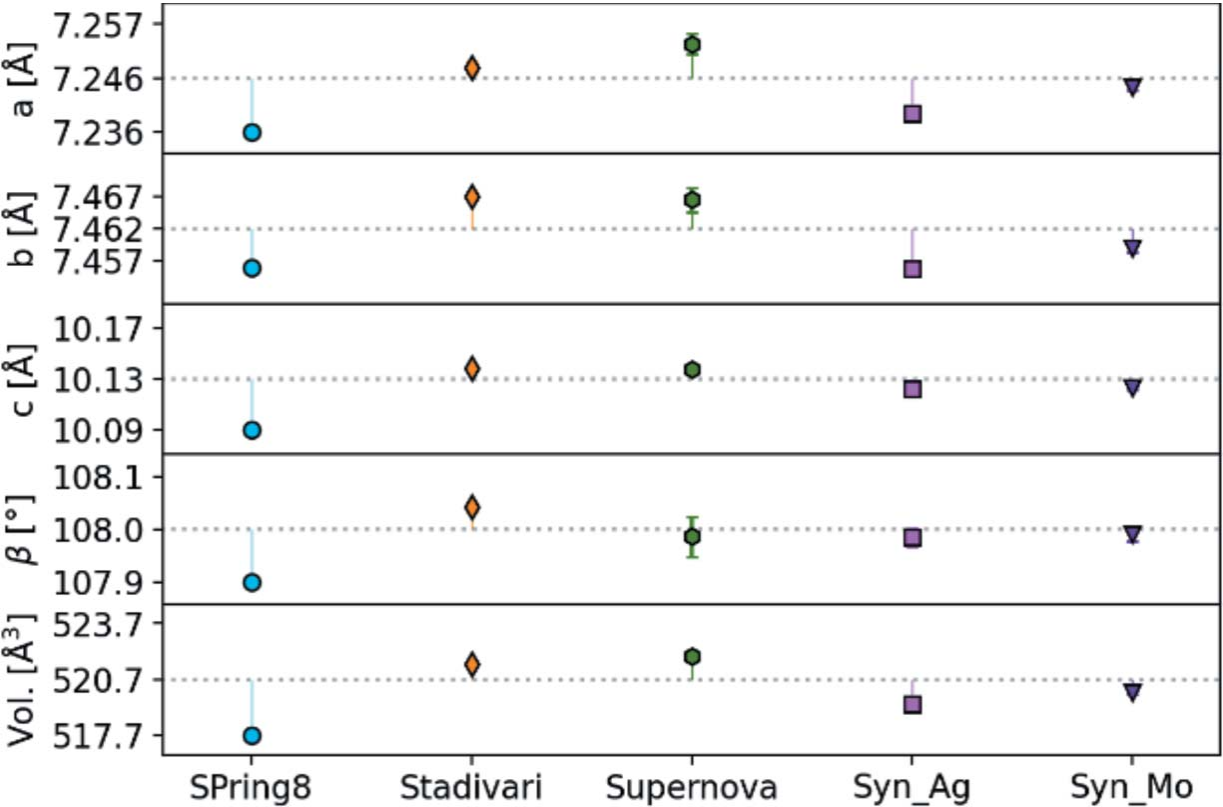
Unit-cell parameters for each dataset. The dotted gray line denotes the average of all 100 K datasets for each parameter. Error bars are covered by the data point markers.

The unit-cell parameters give an initial comparison of the five datasets, and they are plotted in Fig. 3 ▸, and listed in Table 1 ▸. As expected the SPring-8 dataset gives a lower unit-cell volume and *a*, *b*, *c* and β parameters due to the lower temperature of 25 K. All conventional diffractometer data were measured at 100 K and they give unit-cell parameters close to the mean value, with the Supernova giving a slightly higher unit-cell volume of 521.94 (3) Å^3^ and the Synergy_Ag a slightly lower volume of 519.36 (2) Å^3^.

### 3.4. Atomic displacement parameters

**Figure 4:**
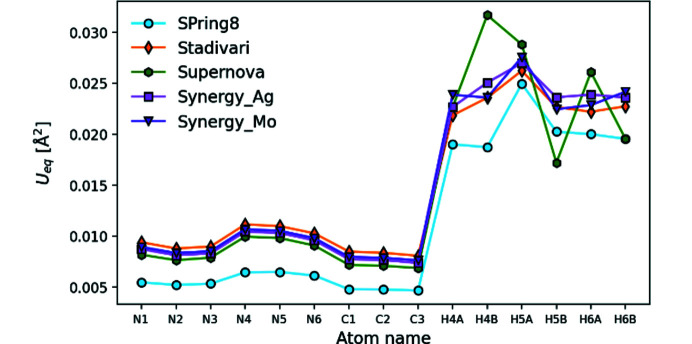
*U*_eq_ found as the average of the trace elements in the ADP matrix. Note that the SPring-8 data were measured at 25 K, whereas the other datasets were measured at 100 K.

Atomic displacement parameters (ADPs) are sensitive probes for systematic errors in crystallographic refinements, and they provide a stringent test on data quality (Iversen *et al.*, 1996 ▸; Morgenroth *et al.*, 2008 ▸). Generally, all datasets follow the same trend for ADPs as seen in Fig. 4 ▸. The ring nitro­gen and carbon atoms vibrate significantly less than the amine nitro­gen, as expected from the very rigid structure of the aromatic system. Hydrogen ADPs obtained from HAR are larger, and differ more between atoms, but follow the same trend for each dataset, with the Supernova *U*_eq_ values diverging considerably. As expected from the temperature difference and noted for the unit-cell parameters in Fig. 3 ▸, the SPring-8 dataset gives consistently lower ADPs than the conventional diffractometers.

We arbitrarily choose Synergy_Ag as the reference dataset, and the mean ratio of *U*_*ii*_ values and mean absolute delta Δ*U*_*ij*_ values then have been calculated using the *UIJXN* program (Blessing, 1995 ▸) and shown in Table 3 ▸. As in Fig. 4 ▸ we generally see a better agreement for all datasets when only *p*-block elements are included in the comparison, as evidence of a lower agreement between hydrogen ADPs. All conventional sources have mean ratio values of *U*_*ii*_ equal to 1 within the standard deviation, meaning that these are in excellent agreement. Δ*U*_*ij*_ values are often calculated between X-ray and neutron data to estimate the level of systematic error in the data and for the best datasets in the literature measured at lowest possible temperature the Δ*U*_*ij*_ values approach ∼0.0002 Å^2^ for non-hydrogen atoms (Morgenroth *et al.*, 2008 ▸). However, for typical liquid nitro­gen temperature datasets the *X*–N agreement is often an order of magnitude larger, and this is also the level of agreement between the individual 100 K datasets in the present study. As a side note, a benchmark study probing the physical soundness of highest quality ADPs for molecular crystals by quantitative comparison with state of the art phonon calculations could be useful, similar to works on inorganic solids (Bindzus *et al.*, 2014 ▸; Beyer *et al.*, 2023 ▸).

### 3.5. Multipole populations

In the Hansen–Coppens multipole formalism the aspherical atomic density, ρ_atom_, is described as:

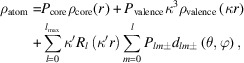

where the multipoles are given by the normalized spherical harmonic functions, *d_lm±_*, and the populations are found by the parameters, *P_lm±_*, with *l* = 0…4 and *m* = 0…*l* for the monopole, dipoles, quadrupoles, octupoles and hexadecapoles, respectively.

**Figure 5:**
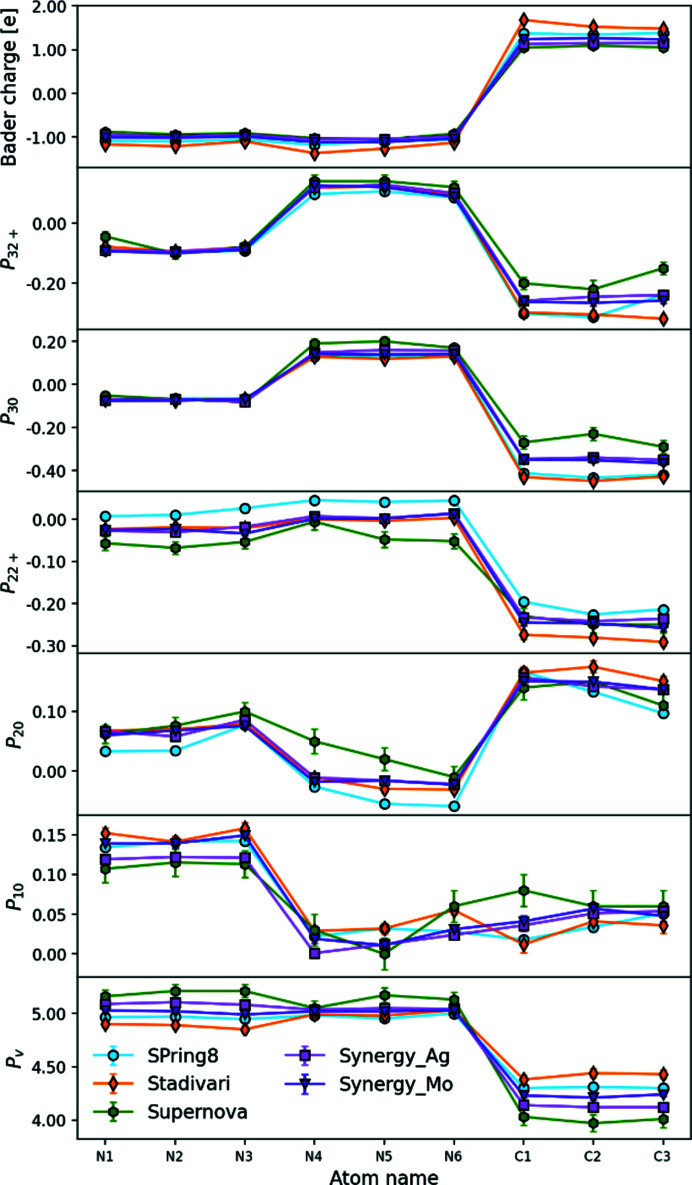
Multipole populations plotted for all *p*-block atoms for each dataset. *P*_v_ is the monopole population, *P*_10_ is the *l* = 1, *m* = 0 dipole parameter, *P*_20_ and *P*_22+_ are the *l* = 2, *m* = 0, 2+ quadrupole parameters, and *P*_30_ and *P*_33+_ are the *l* = 3, *m* = 0, 2+ octupole parameters. The top plot shows the Bader charges.

In the multipole model, the two 1*s* electrons on both carbon and nitro­gen atoms are treated as core electrons, while the remaining valence electrons are allowed to displace with the multipole deformations. The orientation of the multipoles is defined by the choice of individual local coordinate system for each atom. For all *p*-block atoms the *x* axis is defined to be perpendicular to the ring, while the *y* and *z* axes are in the plane of the ring, with the *z* axis pointing towards the center, and the *y* axis to the nearest neighbor (ensuring a right-handed coordinate system). Multipole parameters are shown in Fig. 5 ▸ for the *d*_10_, *d*_20_ and *d*_30_ di-, quadru- and octupoles oriented along the *z* axis in the plane of the ring, and the *d*_22+_ and *d*_32+_ multipoles with lopes perpendicular to the ring.

The bottom graph in Fig. 5 ▸, shows that the monopole population (*P*_v_) for the carbon and nitro­gen atoms have a spread of about 0.5 e. The nitro­gen values spread around the number of electrons in the valence shell (five), whereas the carbon atoms tend to be larger than four signifying negative atoms. In the early electron density literature monopole values were often used to estimate atomic charges in molecular systems (Coppens, 1997 ▸), but negative carbon atoms contradict chemical expectation for the melamine molecule containing electronegative nitro­gen atoms. In addition, the spread of about half an electron among the datasets is chemically unacceptable. However, as shown below, the topological analysis of the multipole densities provides stronger similarity among the datasets and the Bader charges shown in Fig. 5 ▸ only have a spread of about 0.2 e with nitro­gen being negative and carbon being positive. This shows that the total densities are reliable even if they are projected differently into the multipole parameters by the least square procedure when refining the different datasets.

The di-, quadru- and octupole parameters in Fig. 5 ▸ are shown for a selection of the most significant m values of 0 (in plane) and 2+ (perpendicular to the ring). The octupoles on the nitro­gen atoms show remarkable agreement, whereas they differ significantly on the carbon atoms. The dipoles and quadrupoles also have significant spread in values and in general the most extreme values are seen for the Supernova and SPring-8 datasets. The slightly different projections into the multipole parameters nevertheless result in total densities with good agreement as detailed below.

### 3.6. Topology

Bader topological analysis of the electron density is used for quantitative analysis of chemical bonding (Bader, 1994 ▸). Assuming a successful deconvolution of thermal and electronic effects, we expect similar estimates of bonding properties for all datasets regardless of the temperature.

**Figure 6:**
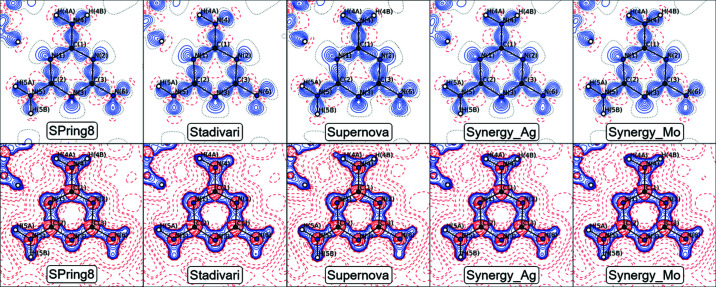
Top: Static deformation density maps (ρ_MM_-ρ_IAM_) with contour intervals of at 0.1 e Å^−3^. Positive (solid blue), zero (dotted black) and negative (broken red) contours. Bottom: Laplacian maps, 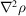 , with logarithmic contour levels. Negative (solid blue), zero (dotted black) and positive (broken red) contours. Both types of maps are calculated in a 2 × 2 × 2 cell. Only atoms within a 0.2 Å distance of the plane defined by C1/C2/C3 are labeled in the plot.

The static deformation density and Laplacian maps in Fig. 6 ▸ show that all models produce similar densities for melamine, and clear nitro­gen lone pairs and covalent bonding densities are obtained for all models. The Supernova deformation density map shows small inconsistencies *e.g.* with overlap of bonding densities and lack of negative contours near the nuclei on electronegative atoms (Poulsen *et al.*, 2007 ▸). Such features typically signify less accurate multipole densities (possibly slight scale factor errors). The best agreement with the SPring-8 data is obtained for the Stadivari and Synergy_Mo datasets. All Laplacian maps show similar features, except H4*A* in the Supernova dataset, which has a questionably elongated electron concentration into the hydrogen bonding region. A similar feature has been observed in very strong hydrogen bonds (Overgaard *et al.*, 1999 ▸), but since these are not present in the melamine crystal, the Supernova dataset in this aspect lead to clearly unphysical features.

**Figure 7:**
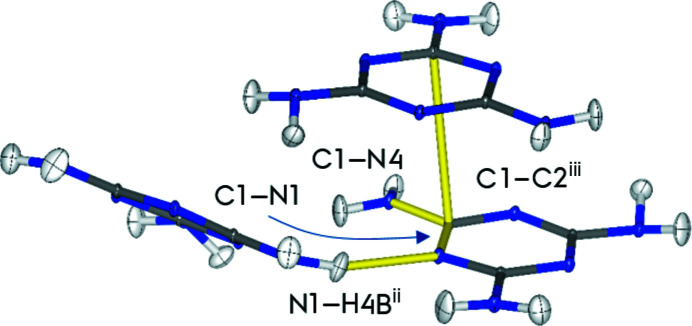
Selected bonds, C1—N1, C1—N4, N1⋯H4*B* ^ii^ and C1⋯C2^iii^, for the bcp analysis are labeled and highlighted in yellow. Superscripts indicate atoms in neighboring molecules, no superscript indicates atoms at position (*x*, *y*, *z*). C2^iii^ is the C2 atom in the melamine molecule at the position (1 − *x*, 1 − *y*, 1 − *z*), while H4*B* ^ii^ is at position (½ − *x*, *y* − ½, *z* − ½).

**Figure 8:**
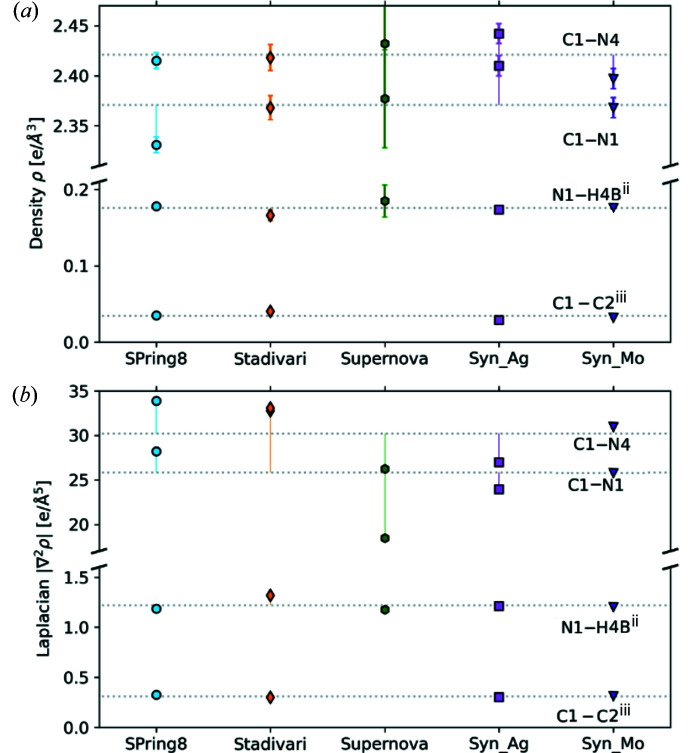
(*a*) Density and (*b*) the absolute value of the Laplacian at different bcps. The bcp labels are given to the right in each figure. The dotted gray lines denote the average of all datasets for each bcp. Laplacian error bars are covered by the data point markers.

For a quantitative comparison of the multipole densities, four bond critical points (bcps) differing in chemical nature (shown in Fig. 7 ▸) are compared in Table 4 ▸. The selected bcps include two intramolecular covalent bonds between carbon and nitro­gen in the ring, and a carbon and an amine nitro­gen, labeled C1—N1 and C1—N4, respectively. The other two bcps are intermolecular interactions, one is a stronger hydrogen bond between N1⋯H4*B* ^ii^ and the other is a weaker C1⋯C2^iii^ π–π interaction. The superscripted number refers to the symmetry operation performed to transfer melamine in the asymmetric unit into the other four molecules in the unit cell. In this case the C1⋯C2^iii^ bcp is between atoms at positions (*x*, *y*, *z*) and (iii: 1 − *x*, 1 − *y*, 1 − *z*), corresponding to an inversion and a translation of 1 along all unit-cell edges. The N1⋯H4*B* ^ii^ bcp is between N1 at (*x*, *y*, *z*) and H4*B* in the molecule at position (ii: ½ − *x*, *y* − ½, *z* − ½), corresponding to the twofold screw axis and a translation of (0, −1, 0). Values for the electron density and the Laplacian for each of the four bcps are plotted in Fig. 8 ▸.

Generally, all topological values agree with the expected values for covalent and intermolecular interactions, according to Gatti (2005 ▸), and only small differences are observed even for the Supernova dataset. One exception is that this dataset does not give a bcp for the weak intermolecular interaction between C1 and C2^iii^, in contrast to the rest of the datasets. For the two covalent C—N bonds the SPring-8 dataset has density and Laplacian values lower and higher, respectively, than the mean values for the two covalent bonds. This is reproduced by the Stadivari and Synergy_Mo datasets corroborating the comparison of fine features in the static deformation densities. In general, the differences in bcp density and Laplacian values between the datasets are significantly larger than the least-squares error, except for the Supernova dataset which has errors five times larger than the other datasets. It is well established that the systematic error in the multipole model is larger than the random least-squares error (Shi *et al.*, 2019 ▸). Table 4 ▸ clearly shows that the systematic error between studies conducted on different crystals and different diffractometers is about an order of magnitude larger than the random least-squares error. The ellipticity is a particularly sensitive parameter being the ratio between second derivatives of the density, and as expected it shows some variation among the studies. However, it is notable that the same chemical conclusions are obtained from all datasets (including the Supernova), *i.e.* a π contribution to the covalent C—N bonds (ɛ ∼ 0.1–0.2), largely isotropic hydrogen bonds (ɛ ∼ 0), and very large ellipticity values for the weak intermolecular interaction (ɛ ranging from 1.71 to 9.17).

### 3.7. 3D difference density maps

**Figure 9:**
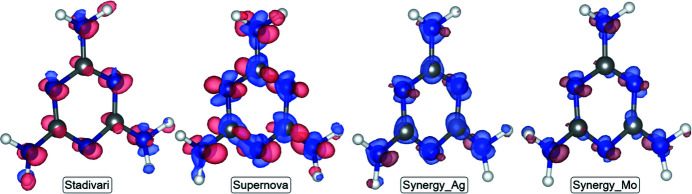
Density difference maps relative to the SPring-8 model. The density is shown at a ±0.1 e Å^−3^ isosurface. Blue is positive and red is negative density.

For a visual comparison of the model electron densities, 3D density difference maps have been calculated, to visualize where the models differ. The SPring-8 dataset is chosen as the reference dataset for the comparison. All difference maps are calculated by subtracting the SPring-8 ED data from the other ED data, and the results are visualized in Fig. 9 ▸ at a ±0.1 e Å^−3^ isosurface level.

In line with the conclusions above the best agreement with the SPring-8 model is obtained for Stadivari and Synergy_Mo. The Synergy_Mo density agrees particularly well with the SPring-8 density, whereas the Supernova has significantly poorer agreement. For the Supernova difference density the most significant features are the negative areas perpendicular to the plane of the ring. The lack of density perpendicular to the ring in the Supernova dataset may explain the missing C⋯C bcp in the topological analysis. Clearly, the modern laboratory diffractometers represent a significant improvement in data quality over the aging Supernova, and subtle (possibly important) density features are much better revealed.

## 4. Conclusion

In this study, we have compared the data quality and derived multipole model electron density of four conventional X-ray diffractometer sources (100 K) and one synchrotron dataset (25 K). The synchrotron dataset has a significantly higher number of reflections (higher resolution of 1.45 Å^−1^), higher 〈*I*/σ〉, and higher redundancy. This makes it possible to refine more subtle electron features such as hydrogen kappa parameters. However, the conventional datasets still have excellent resolution (∼1.20 Å^−1^) and the final multipolar models are of comparable very high quality in the case of the modern Stadivari and Synergy diffractometers. The dataset from the aging Supernova diffractometer is clearly more noisy, but it is possible to obtain a quite reasonable electron density for melamine. However, it does contain small spurious deformation density features and lack of a specific weak intermolecular bcp. The Stadivari and Synergy-S conventional diffractometers give multipole densities that compare very well with the SPring-8 result showing that an adequate deconvolution of thermal motion can be obtained at 100 K for the molecular crystal of melamine. In the very subtle density features, there is a tendency that the Stadivari and Synergy data measured with Mo *K*α radiation agree slightly better with the SPring-8 model. Significant differences are observed between the refined multipole parameters in the individual models, whereas the total densities show excellent agreement. The present study confirms that monopole populations are not good proxies for atomic charges, and chemical conclusions are better based on topological analysis of the total density. The systematic error between the five densities is about an order of magnitude larger than the random error estimated from the individual least-squares refinements, and thus experimental electron density studies should be prudent when interpreting and reporting errors on densities or Laplacian values at bond critical points. We conclude that for good quality crystals of small organic molecules such as melamine, experimental multipole model electron densities can be obtained at 100 K using modern conventional diffractometers with a quality that approaches, but not quite reaches, results from very high-resolution 25 K synchrotron data. However, in cases with poorly scattering crystals, significant anharmonicity or significant absorption and extinction effects, the short wavelength and high intensity of the synchrotron beam as well as helium cooling are probably needed to provide reliable data.

## Supplementary Material

Crystal structure: contains datablock(s) 100K_Stadivari, 100K_Supernova, 100K_Synergy_Ag, 100K_Synergy_Mo. DOI: 10.1107/S2052520623006625/px5057sup1.cif

Structure factors: contains datablock(s) XD. DOI: 10.1107/S2052520623006625/px5057100K_Stadivarisup2.hkl

Structure factors: contains datablock(s) XD. DOI: 10.1107/S2052520623006625/px5057100K_Supernovasup3.hkl

Structure factors: contains datablock(s) XD. DOI: 10.1107/S2052520623006625/px5057100K_Synergy_Agsup4.hkl

Structure factors: contains datablock(s) XD. DOI: 10.1107/S2052520623006625/px5057100K_Synergy_Mosup5.hkl

Supporting information file. DOI: 10.1107/S2052520623006625/px5057sup6.pdf

Supporting information file. DOI: 10.1107/S2052520623006625/px5057100K_Stadivarisup7.cml

CCDC references: 2267921, 2267922, 2267923, 2267924

## Figures and Tables

**Table 1:** Crystallographic and experimental information of the X-ray diffraction experiments For all experiments: C_3_H_6_N_6_, *M*_r_ = 126.12 g mol^−1^, monoclinic, *P*2_1_/*n*, *Z* = 4, *F*(000) = 264.

	SPring-8	Stadivari	Supernova	Synergy_Ag	Synergy_Mo
Crystal dimensions (µm)	140 × 100 × 100	190 × 140 × 100	200 × 160 × 100	200 × 160 × 100	200 × 160 × 100
Wavelength (Å)	0.248	0.71 (Mo)	0.71 (Mo)	0.56 (Ag)	0.71 (Mo)
Temperature (K)	25 (2)	100 (2)	100 (2)	100 (2)	100 (2)
Maximum sinθ/λ (Å^−1^)	1.45	1.17	1.17	1.25	1.25
*a* (Å)	7.2355 (2)	7.2484 (12)	7.253 (2)	7.2392 (13)	7.2445 (7)
*b* (Å)	7.4554 (2)	7.4668 (7)	7.466 (2)	7.4552 (11)	7.4584 (6)
*c* (Å)	10.0871 (3)	10.1361 (11)	10.135 (4)	10.1192 (17)	10.1200 (13)
β (°)	107.9240 (13)	108.073 (10)	108.01 (4)	108.012 (19)	108.018 (12)
Volume (Å^3^)	517.728 (35)	521.52 (12)	521.94 (3)	519.363 (15)	519.998 (9)
ρ (g cm^−3^)	1.618	1.606	1.605	1.613	1.611
μ (mm^−1^)	0.033	0.12	0.12	0.074	0.12
Collection time (min)	25	3228	1963	3416	1513
Exposure time (low/high detector angles) (s)	0.14	7/50	10/80	90	7/40
Scan width (° per frame)	0.1	0.5	1.0	0.5	0.5
Detector distance (mm)	130	60	52	40	40
Beam FWHM (µm)	n/a	120	150	100	120
*R*_int_ (%)	3.97	2.67	10.5	3.02	3.03
Completeness (%)†	100.0	99.9	100.0	100.0	100.0
〈*I*/σ〉†	43.97	41.82	13.48	32.33	34.31
Redundancy†	15.9	6.7	7.5	7.8	8.1
*N*_measured_	164296	46567	52393	61069	61701
*N*_unique_ ‡	13231	6337	6878	7931	7537

† The values for 〈*I*/σ〉, completeness and redundancy are listed for a resolution cutoff of 1.17 Å^−1^, which is the highest common resolution for all datasets. All other parameters are given to full resolution of the individual datasets.

‡ *N*_unique_ is the number of unique reflections measured more than three times.

**Table 2:** Information for quality control of the multipole models for each dataset Values with both an *I* > 3σ and no *I*/σ cutoff are included, separated by a slash. Numbers in parenthesis are associated with the refinement using *I* > 3σ and data up to 1.17 Å^−1^ for comparison, while all other values in this table are given for the full resolution range [max sinθ/λ (Å^−1^)]. *N*_ref_/*N*_v_ is the number of reflections used in the refinement, *N*_ref_, divided by the number of parameters refined in the model, *N*_v_. *N*_rejected_ is the number of reflections omitted due to the *I* > 3σ criterion.

	SPring-8	Stadivari	Supernova	Synergy_Ag	Synergy_Mo
Max sinθ/λ (Å^−1^)	1.45 (1.17)	1.17/1.17	1.17/1.17	1.25/1.25 (1.17)	1.25/1.25 (1.17)
*R*(*F*) (%)	1.36 (0.96)	1.37/3.14	2.92/8.16	1.46/2.56 (1.33)	1.33/2.05 (1.26)
*R*(*F* ^2^) (%)	1.64 (1.38)	1.65/1.89	3.37/4.22	1.60/1.69 (1.52)	1.65/1.71 (1.59)
GOF	1.087 (1.148)	1.183/1.082	0.990/0.917	1.016/0.955 (1.013)	1.023/0.972 (1.012)
*N*_ref_	11027 (6498)	4961/6337	4327/7052	6351/7930 (5646)	6299/7536 (5612)
*N*_rejected_	2132 (628)	1376/0	2648/0	1579/0 (1185)	1237/0 (1004)
*N*_ref_/*N*_v_	33.42 (19.69)	15.03/19.20	13.11/21.37	19.25/24.03 (17.11)	19.09/22.84 (17.01)
Gross residual (e Å^−3^)	0.028 (0.020)	0.025/0.042	0.057/0.097	0.027/0.037 (0.024)	0.025/0.032 (0.024)
Min residual (e Å^−3^)	−0.12 (−0.09)	−0.10/−0.21	−0.25/−0.40	−0.13/−0.15 (−0.11)	−0.12/−0.14 (−0.11)
Max residual (e Å^−3^)	0.21 (0.14)	0.11/0.18	0.25/0.39	0.10/0.14 (0.09)	0.09/0.12 (0.09)
Correlation coefficient	0.77: κ(C)/*M*1 on C2	0.70: κ(C)/*M*1 on C1 and C3	0.77: *M*1 on N1 and C1	0.77: *M*1 on C2 and C3	0.72: κ(C)/*M*1 on C1, C2 and C3

**Table 3:** Mean ratio of *U*_*ii*_ values and mean absolute Δ*U*_*ij*_ values for each dataset compared to Synergy_Ag Estimated standard deviation from the mean is given in the parenthesis. Note that the SPring-8 data were measured at 25 K, whereas the other datasets were measured at 100 K.

Data set	Elements included	Mean ratio of *U*_*ii*_ values, 〈*U*_*ii*_(*X*)/*U*_*ii*_(Syn_Ag)〉	Mean absolute Δ*U*_*ij*_
SPring-8	All	0.7 (2)	0.002 (2)
	*p*-block	0.63 (2)	0.002 (2)
Stadivari	All	1.0 (1)	0.003 (4)
	*p*-block	1.08 (2)	0.001 (1)
Supernova	All	1.0 (3)	0.003 (4)
	*p*-block	0.93 (7)	0.0004 (4)
Synergy_Mo	All	1.02 (7)	0.002 (4)
	*p*-block	1.03 (2)	0.001 (1)

**Table 4:** Topological analysis of the electron density for all datasets showing the selected intramolecular C1—N1 and C1—N4, as well intermolecular N1⋯H4*B* ^ii^ and C1⋯C2^iii^ bcps *R* is the distance between the atoms along the bond path, *d*1 and *d*2 are the distances from the first mentioned atom to the bcp and from the bcp to the latter atom, respectively. ρ is the density, ∇^2^ρ is the Laplacian and ɛ is the bond ellipticity. *G*, *V* and *E* are the kinetic, potential and total energies, respectively, given in Hartree Å^−3^ (H Å^−3^).

	Bond	*R*	*d*1 (Å)	*d*2 (Å)	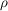 (e Å^−3^)	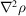 (e Å^−5^)	ɛ	*G* (H Å^−3^)	*V* (H Å^−3^)	*E* (H Å^−3^)	|*V*|/*G*
SPring-8	C1—N1	1.3470	0.5503	0.7967	2.331 (8)	−28.23 (4)	0.21	1.98	−5.93	−3.95	3.00
	C1—N4	1.3387	0.5114	0.8273	2.415 (8)	−33.90 (4)	0.27	1.91	−6.20	−4.29	3.24
	N1⋯H4*B* ^ii^	2.0509	1.2946	0.7563	0.178 (4)	1.186 (2)	0.01	0.10	−0.12	−0.02	1.18
	C1⋯C2^iii^	3.4828	1.7216	1.7612	0.035 (1)	0.325 (1)	2.89	0.02	−0.02	0.00	0.75
Stadivari	C1—N1	1.3490	0.5311	0.8179	2.37 (1)	−32.78 (6)	0.12	1.85	−6.00	−4.15	3.24
	C1—N4	1.3388	0.4527	0.8861	2.42 (1)	−33.08 (8)	0.11	1.96	−6.23	−4.27	3.18
	N1⋯H4*B* ^ii^	2.0418	1.3143	0.7274	0.166 (7)	1.319 (3)	0.04	0.10	−0.11	−0.01	1.09
	C1⋯C2^iii^	3.4838	1.7488	1.7350	0.040 (1)	0.299 (1)	1.71	0.02	−0.02	0.00	0.82
Supernova	C1—N1	1.3470	0.6209	0.7261	2.38 (5)	−18.5 (2)	0.20	2.54	−6.38	−3.84	2.50
	C1—N4	1.3387	0.5563	0.7825	2.43 (6)	−26.3 (3)	0.30	2.31	−6.46	−4.15	2.80
	N1⋯H4*B* ^ii^	2.0534	1.2956	0.7578	0.19 (2)	1.177 (7)	0.06	0.10	−0.12	−0.02	1.20
	C1⋯C2^iii^	–	–	–	–	–	–	–	–	–	–
Synergy_Ag	C1—N1	1.3466	0.5917	0.7550	2.41 (1)	−23.97 (5)	0.14	2.36	−6.41	−4.04	2.71
	C1—N4	1.3383	0.5664	0.7719	2.44 (1)	−26.98 (5)	0.23	2.30	−6.49	−4.19	2.82
	N1⋯H4*B* ^ii^	2.0606	1.3058	0.7549	0.174 (5)	1.213 (2)	0.06	0.10	−0.12	−0.02	1.15
	C1⋯C2^iii^	3.4805	1.7387	1.7418	0.029 (1)	0.302 (1)	9.17	0.02	−0.01	0.00	0.70
Synergy_Mo	C1—N1	1.3469	0.5764	0.7705	2.38 (1)	25.78 (4)	0.13	2.22	−6.24	−4.02	2.81
	C1—N4	1.3388	0.5371	0.8017	2.40 (1)	−30.96 (5)	0.21	2.01	−6.18	−4.17	3.08
	N1⋯H4*B* ^ii^	2.0562	1.3101	0.7461	0.176 (5)	1.201 (2)	0.05	0.10	−0.12	−0.02	1.16
	C1⋯C2^iii^	3.4927	1.7346	1.7581	0.032 (1)	0.312 (1)	4.55	0.02	−0.01	0.00	0.73

Symmetry codes: (ii) ½ − *x*, *y* − ½, *z* − ½; (iii) 1 − *x*, 1 − *y*, 1 − *z*.
