# Supplementary material for: Comparative study of conventional and synchrotron X-ray electron densities on molecular crystals
Source: Acta Crystallogr B Struct Sci Cryst Eng Mater. 2023 Sep 4;79(Pt 5):380–91. doi: 10.1107/S2052520623006625 (PMC10552600; doi:10.1107/S2052520623006625)
Supplement: Supplementary file 6 [file b-79-00380-sup6.pdf]

Supporting Information for

Comparative study of conventional and synchrotron X-ray electron densities on molecular crystals

Emilie S. Vosegaard, Jakob V. Ahlburg, Lennard Krause and Bo B. Iversen*

Center for Integrated Materials Research, Department of Chemistry and Interdisciplinary Nanoscience Center (iNANO), Aarhus University, DK-8000 Aarhus C, Denmark

*Corresponding author: bo@chem.au.dk

Crystals used for in-house experiments:

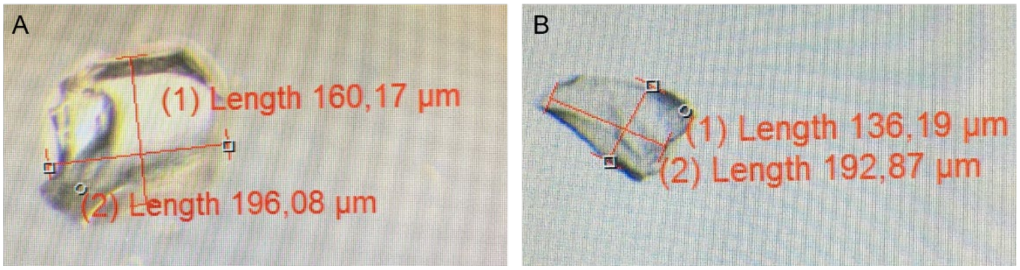

Figure S1. The crystal seen in figure S1A, with dimensions 200x160x100 µm, was used for the in-house experiments at the Synergy-S and Supernova diffractometers. The crystal in Figure S1B was used for the Stadivari experiment, and has the dimensions 190x140x100 µm.

Diffraction experiment run lists:

All angles are given in degrees [˚].

SPring8 25K:

ω scans at different 2θ and chi. 0.1 ˚/frame. Exposure time 0.14 s. Detector distance 130 mm.

Run nr	2θ	χ	ω start	ω end
1	0	0	0	180
2	0	-20	0	180
3	0	-40	0	180
4	20	0	0	180
5	20	-20	0	180
6	20	-40	0	180

Stadivari:

ω scans at different 2θ, χ and φ angles. 0.5 ˚/frame. Exposure time as given in the table. Detector distance 60 mm.

Run nr	2θ	Exp. Time [s]	χ	φ	ω start	ω end
1	-47.4	7	-32	55	120	184
2	-47.4	7	-47	105	184	120
3	-63.5	50	-20	135	107	186
4	-63.5	50	-35	-175	186	107
5	-79.5	50	-37	-160	86	186
6	-79.5	50	-22	150	186	86
7	-79.5	50	-20	-20	91	190
8	-79.5	50	-35	30	190	91
9	-79.5	50	-29	60	93	188
10	-79.5	50	-54	10	188	93
11	-79.5	50	-52	110	106	189
12	-79.5	50	-27	160	188	106
13	-79.5	50	-24	-125	107	188
14	-79.5	50	-39	-75	188	107
15	-79.5	50	-30	-15	95	175
16	-79.5	50	-45	35	175	95
17	-79.5	50	-35	85	111	138
18	-79.5	50	-60	35	138	111
19	-79.5	50	-28	145	87	99
20	-79.5	50	-43	-165	99	87
21	-79.5	50	-52	-110	176	186
22	0.8	7	-55	175	10	-24
23	0.8	7	-40	125	-24	10
24	0.8	7	-28	-30	-5	-18
25	0.8	7	-43	20	-18	-6
26	16.9	7	-56	-140	25	-18
27	16.9	7	-31	-90	-18	25
28	49.0	7	-36	-100	63	-16
29	49.0	7	-51	-50	-16	63
30	74.5	50	-35	55	84	-25
31	74.5	50	-60	5	-25	84
32	74.5	50	-22	30	81	-23
33	74.5	50	-37	80	-23	81
34	74.5	50	-52	130	81	-17
35	74.5	50	-41	35	-19	-1
36	74.5	50	-26	-15	-1	-19

Supernova:

ω scans at different detector, κ and φ angles. 1 ˚/frame. Exposure times as given in the table. Detector distance 52 mm.

Run nr	θ	Exp time [s]	κ	φ	ω start	ω end
1	-42.3	10	-71	76	-114	-12
2	-42.3	10	71	-152	-72	29
3	42.3	10	71	-152	12	114
4	90.7	80	-71	76	19	121
5	90.7	80	71	-152	61	158
6	42.3	10	-125	-60	6	97
7	90.7	80	-125	-60	19	113
8	90.7	80	77	-60	82	158
9	90.7	80	77	-90	82	158
10	90.7	80	77	-120	82	158
11	90.7	80	-30	90	16	115
12	90.7	80	77	120	82	158
13	90.7	80	125	120	76	158
14	42.3	10	57	60	16	112
15	42.3	10	57	-30	16	112
16	42.3	10	57	120	16	112
17	90.7	80	-77	-60	17	122
18	90.7	80	45	30	67	158
19	90.7	80	61	-180	63	158
20	90.7	80	45	90	67	94

Synergy Ag

ω scans at different κ and φ angles. Detector at 38˚. 0.5 ˚/frame. Exposure time 90s. Detector distance 40 mm.

Run nr	κ	φ	ω start	ω end
1	91	15	-75	20
2	-91	-97	-2	23
3	178	-150	43	68
4	178	-150	78	111
5	-178	-60	45	71
6	65	-20	15	70
7	65	-20	82	108
8	-57	0	-18	8
9	-52	158	-6	38
10	-33	66	-11	15
11	-178	90	53	112
12	-36	168	-39	14
13	30	-22	47	81

14	30	-22	92	118
15	-57	-150	-35	-6
16	-4	-77	-38	-12
17	-4	-119	-26	0
18	-19	-150	-39	4
19	63	-126	32	58
20	63	-126	68	94
21	-99	90	-11	15
22	-99	90	47	72
23	-34	-82	-18	9
24	-38	-120	-15	20
25	-38	0	-42	-5
26	-38	30	-30	3
27	-39	147	-42	-13
28	-57	-30	-34	-8
29	-57	30	-4	22
30	57	-90	81	107
31	55	-46	18	49
32	38	60	59	98
33	-19	90	-43	5

Synergy Mo

ω scans at different detector, κ and φ angles. 0.5 ˚/frame. Exposure time as given in the table. Detector distance 40 mm.

Run nr	Detector	Exp time [s]	κ	φ	ω start	ω end
1	-49.6	7	100	-87	-80	22
2	49.8	7	-100	166	0	93
3	-77.7	40	-100	166	-151	-85
4	-77.7	40	100	-87	-81	-4
5	77.9	40	-100	166	5	106
6	95.2	40	100	-87	107	169
7	95.2	40	-100	166	22	106
8	-49.6	7	19	90	-27	31
9	-49.6	7	-125	60	-125	-73
10	-49.6	7	-125	-60	-125	-73
11	-77.7	40	19	30	-27	3
12	-77.7	40	19	60	-27	3
13	77.9	40	19	120	-27	3
14	77.9	40	125	-60	95	154
15	77.9	40	-125	60	34	111
16	77.9	40	125	30	95	154
17	77.9	40	-38	-150	-2	43

18	95.2	40	-38	-180	15	43
19	95.2	40	-57	-180	18	66
20	95.2	40	-38	-60	15	43
21	95.2	40	125	90	95	171
22	95.2	40	-38	0	15	43
23	95.2	40	-57	-120	18	66
24	95.2	40	-38	-30	15	43
25	-49.6	7	-77	90	-119	-21
26	-49.6	7	-77	-180	-119	-21
27	-49.6	7	-77	-90	-119	-45
28	-49.6	7	-77	-30	-119	-21

Test for I/σ cutoff:

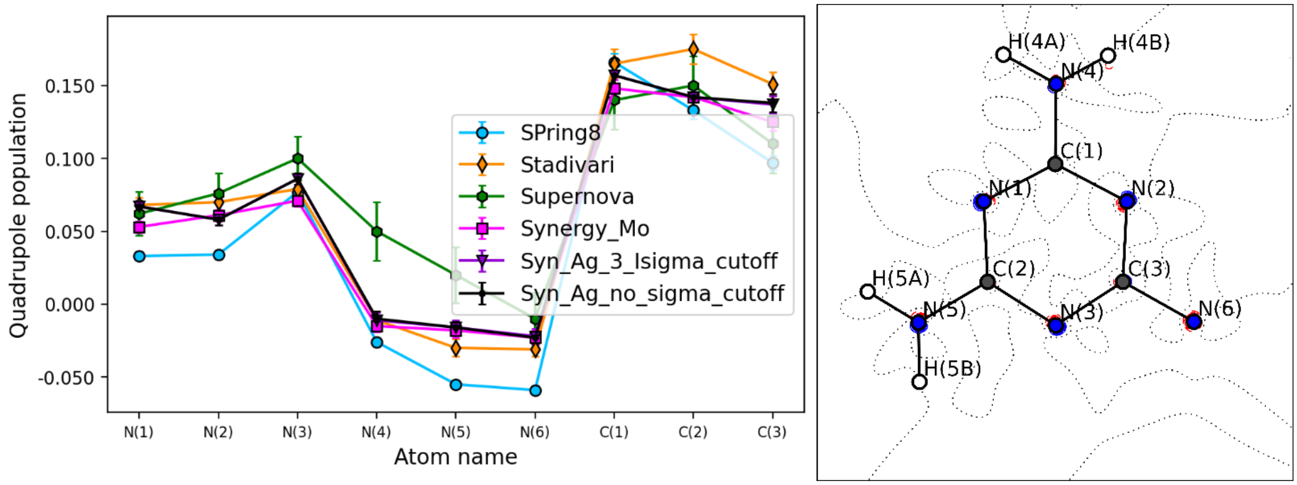

Figure S2. Quadrupole populations and difference density plot (calculated as $\rho[3\ \mathrm{I}/\sigma] - \rho[\mathrm{no}\ \mathrm{I}/\sigma]$) of the Synergy Ag dataset showing the difference when using no I/σ cutoff, and a $\mathrm{I} > 3\sigma$ intensity cutoff. The black dotted line in the difference density plot is zero, red and blue lines are positive and negative features, respectively. Contours are shown at 0.01 e/Å^3 intervals.

The changes in multipole parameters and the derived electron density when using different I/σ cutoffs are negligible and significantly smaller than the differences between datasets, as exemplified by the quadrupole populations in Figure S2. We use a $\mathrm{I} > 3\sigma$ intensity cutoff in this study to only include the most reliable reflections.

Test for 3λ contamination:

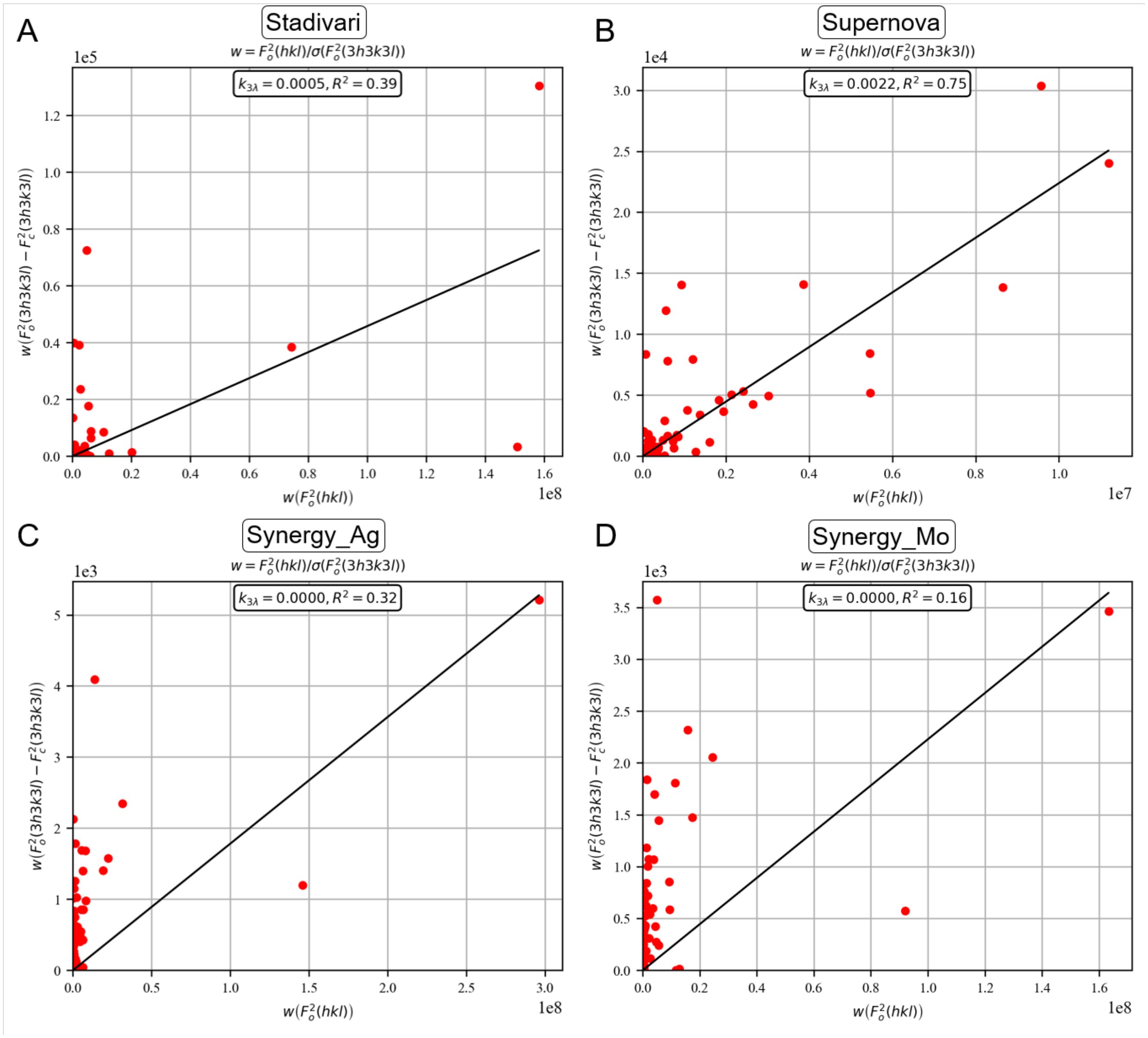

Figure S3. Difference between observed and calculated squared structure factors for reflections with *3h3k3l* indices shown as a function of the corresponding *hkl* for A) Stadivari, B) Supernova, C) Synergy Ag and D) Synergy Mo. The structure factor files for all conventional datasets have been tested for 3-λ contribution as described in (Krause *et al.*, 2015) and only a slight contamination for the Supernova data was found. The modern diffractometers seem to be much less affected. Consequently, as the source setup is very similar to the ones studied in the publication, we propose the single photon counting operation of the employed detectors (EIGER2 and HyPix) to more efficiently suppress the impact of the high energy photons.

Bader charges:

Bader charges of each atom in units of e.

	SPring8	Stadivari	Supernova	Synergy_Ag	Synergy_Mo
N(1)	-1.086	-1.165	-0.8772	-0.9458	-1.005
N(2)	-1.101	-1.209	-0.9376	-0.9796	-1.013
N(3)	-1.056	-1.096	-0.9105	-0.9420	-0.9832
N(4)	-1.177	-1.365	-1.024	-1.043	-1.111
N(5)	-1.084	-1.262	-1.052	-1.041	-1.111
N(6)	-1.068	-1.127	-0.9228	-0.9733	-1.030
C(1)	1.374	1.678	1.043	1.128	1.241
C(2)	1.346	1.521	1.090	1.149	1.260
C(3)	1.380	1.480	1.054	1.156	1.237

References

Krause, L., Herbst-Irmer, R. & Stalke, D. (2015). *J. Appl. Crystallogr.* **48**, 1907-1913.
